# Voluntary aerobic exercise increases arterial resilience and mitochondrial health with aging in mice

**DOI:** 10.18632/aging.101099

**Published:** 2016-11-22

**Authors:** Rachel A. Gioscia-Ryan, Micah L. Battson, Lauren M. Cuevas, Melanie C. Zigler, Amy L. Sindler, Douglas R. Seals

**Affiliations:** ^1^ Department of Integrative Physiology, University of Colorado Boulder, Boulder, CO 80309, USA

**Keywords:** exercise, vascular function, mitochondria

## Abstract

Mitochondrial dysregulation and associated excessive reactive oxygen species (mtROS) production is a key source of oxidative stress in aging arteries that reduces baseline function and may influence resilience (ability to withstand stress). We hypothesized that voluntary aerobic exercise would increase arterial resilience in old mice. An acute mitochondrial stressor (rotenone) caused greater (further) impairment in peak carotid EDD in old (~27 mo., OC, n=12;−32.5±-10.5%) versus young (~7 mo., YC n=11;−5.4±- 3.7%) control male mice, whereas arteries from young and old exercising (YVR n=10 and OVR n=11, 10-wk voluntary running;−0.8±-2.1% and −8.0±4.9%, respectively) mice were protected. *Ex-vivo* simulated Western diet (WD, high glucose and palmitate) caused greater impairment in EDD in OC (-28.5±8.6%) versus YC (-16.9±5.2%) and YVR (-15.3±2.3%), whereas OVR (-8.9±3.9%) were more resilient (not different versus YC). Simultaneous *ex-vivo* treatment with mitochondria-specific antioxidant MitoQ attenuated WD-induced impairments in YC and OC, but not YVR or OVR, suggesting that exercise improved resilience to mtROS-mediated stress. Exercise normalized age-related alterations in aortic mitochondrial protein markers PGC-1α, SIRT-3 and Fis1 and augmented cellular antioxidant and stress response proteins. Our results indicate that arterial aging is accompanied by reduced resilience and mitochondrial health, which are restored by voluntary aerobic exercise.

## INTRODUCTION

Cardiovascular diseases (CVD) are the leading cause of death in developed societies [1]. The risk of CVD increases progressively with advancing age, such that greater than 90% of deaths from CVD occur in people over the age of 55 [2]. Although the mechanisms underlying the age-related increase in CVD risk have not been fully elucidated, strong evidence indicates that the development of arterial dysfunction is a key factor [3, 4]. An important manifestation of arterial dysfunction is vascular endothelial dysfunction, characterized by a decline in endothelium-dependent dilation (EDD) [5, 6, 7].

A major mechanism underlying the development of age-related endothelial dysfunction is oxidative stress, characterized by excessive production of reactive oxygen species (ROS) relative to endogenous antioxidant defense capacity. Oxidative stress can disrupt many aspects of arterial function, including reducing the bioavailability of the vasodilatory and vasoprotective molecule nitric oxide (NO), resulting in impaired EDD [5–9]. A key source of arterial oxidative stress is excessive production of mitochondrial reactive oxygen species (mtROS). Whereas healthy mitochondria are critical mediators of arterial homeostasis [10–14] and produce physiological levels of mtROS vital for cell signaling [15], declines in mitochondrial health are characterized by excessive mtROS production [10–12, 15–16]. We have recently shown that excess arterial mtROS production is a major contributor to tonic arterial oxidative stress-mediated suppression of EDD with primary aging in mice [13].

Emerging evidence suggests that, in addition to baseline deficits in vascular function, aging may also be accompanied by reduced arterial resilience, i.e., the ability to withstand stress. Aging exacerbates the effects of common *in vivo* stressors such as a “Western”-style (high fat/high sugar) diet, hyperglycemia, and elevated low-density lipoprotein (LDL) cholesterol, such that the age- and stressor-associated impairments of arterial function are compounded, resulting in a greater degree of impairment [17–20]. Because human aging occurs in the presence of numerous stressors, it is important to understand how aging alters arterial resilience and to identify potential interventions that may improve the ability of arteries to withstand these challenges.

Mitochondria are critical components of the cellular stress response and interact with and regulate other stress response mediators, including antioxidant enzymes and heat shock proteins (Hsp) [21–25]. Thus, mitochondrial dysregulation has the potential to impact major upstream mechanisms, such as oxidative stress, that mediate vascular function [26]. However, it is unknown whether age-related declines in arterial mitochondrial health contribute to decreased resilience in the presence of acute stressors.

Aerobic exercise is a powerful intervention that improves baseline endothelial function in the setting of aging [17, 30–33]. It is well known that aerobic exercise improves mitochondrial biogenesis and homeostasis in non-vascular tissues [34–39], and recent work suggests that exercise can also improve markers of arterial mitochondrial content and health in healthy animals [27–28, 40–42], but the effects of aerobic exercise on arterial mitochondria with primary aging are unclear. We tested the hypothesis that aging would be associated with impaired arterial resilience to acute stress and reduced arterial mitochondrial health in mice, and that voluntary aerobic exercise initiated in late-life (10 weeks of voluntary wheel running) would increase resilience and improve mitochondrial health in aging arteries.

## RESULTS

### Morphological characteristics and voluntary wheel running

General morphological characteristics and running wheel activity are presented in Table I. Body mass did not differ among groups following the 10-week voluntary aerobic exercise intervention and age-related changes in heart mass (increase), visceral fat mass (decrease) and muscle mass (decrease) were unaltered by the late-life voluntary aerobic exercise intervention, similar to our previous reports [17, 33]. Carotid artery diameter was increased with aging and with voluntary aerobic exercise. Voluntary running activity was significantly greater in young versus old mice, but the average daily running activity in the old voluntary running group was similar to levels previously reported by our laboratory to improve arterial function in old mice [17, 33].

**Table I:** Select morphological characteristics and voluntary running wheel activity

	YC	OC	YVR	OVR
Body mass (g)	32.15 (2.94)	30.71 (3.12)	32.08 (4.0)	28.95 (1.77)
Heart mass (mg)	152.9 (10.3)	193.0 (18.2)*^^^	144.6 (11.6)	216.5 (4.6)*^^^
Liver mass (g)	1.81 (0.15)	1.65 (0.24)	1.58 (0.33)	1.69 (0.16)
Quadriceps mass (mg)	198.4 (36.3)	143.3 (21.5)*^^^	190.3 (16.8)	138.4 (9.23)*^^^
Visceral fat mass (mg)	82.49 (48.1)	24.4 (13.3)*^^^	85.6 (60.2)	12.2 (6.8)*^^^
Carotid artery diameter (μm)	418 (18)^#^	448 (20)*^#^	451 (20)*	470 (40)*
Running activity (km/day)	n/a	n/a	19.37 (14.0)	3.13 (1.80)^^^

Data are presented as means (SD), n=10-12/group.Abbreviations: YC, young control mice; OC, old control mice; YVR, young voluntary wheel running mice; OVR, old voluntary wheel running mice.

* p<0.05 vs. YC;

^ p<0.05 vs. YVR

# p<0.05 vs. OVR

### Voluntary aerobic exercise reverses vascular endothelial dysfunction and normalizes arterial mitochondrial superoxide production in old mice

**Figure 1:**
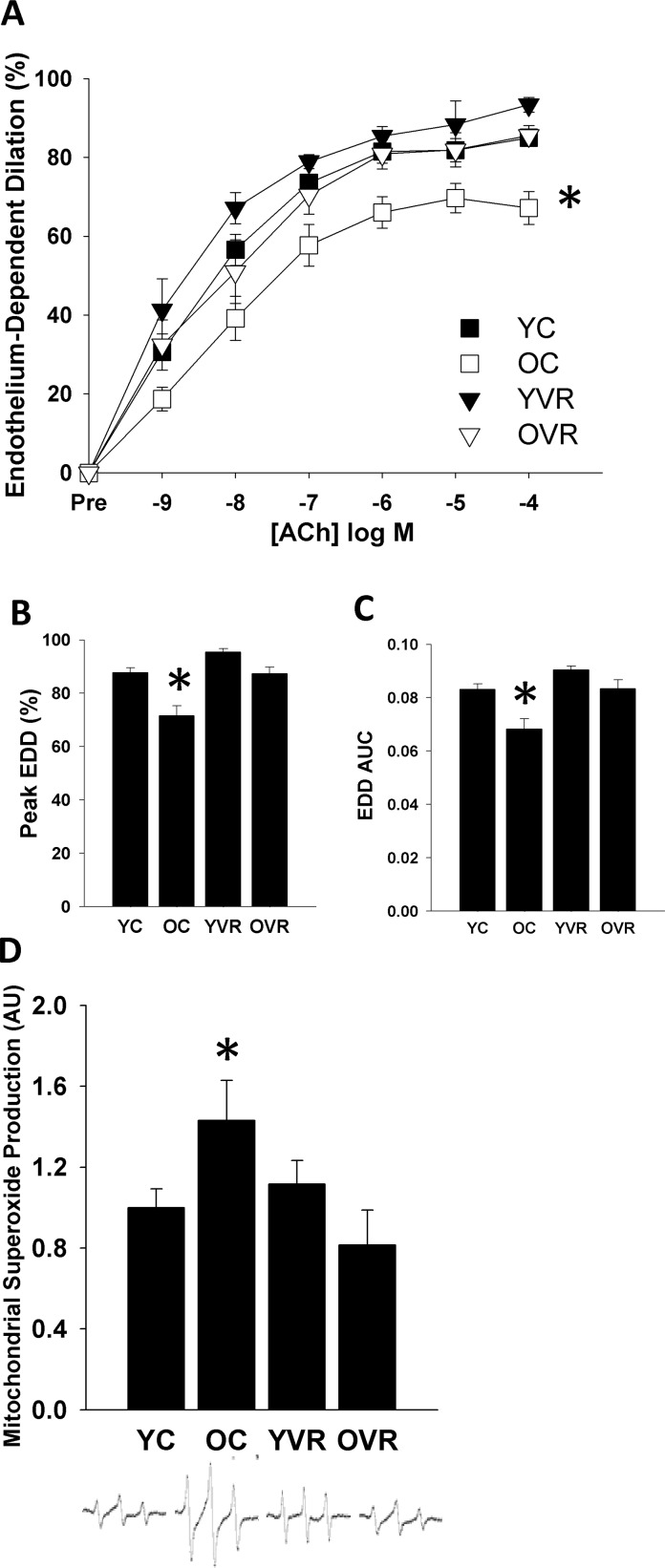
Voluntary aerobic exercise restores endothelium-dependent dilation and normalizes mitochondrial superoxide production in old mice Endothelium-dependent dilation (EDD) dose-response (**A**) to acetylcholine (ACh), peak dilation (**B**), and EDD AUC (**C**) in carotid arteries and aortic mitochondria-specific superoxide production (**D**) in young control (YC), old control (OC), young voluntary wheel running (YVR) and old voluntary wheel running (OVR) mice. Representative EPR spectra presented below panel **D**. Data are presented as means with error bars representing SEM, n=10-12 per group. * p<0.05 vs. all other groups. Peak EDD and EDD AUC data are shown again in Figures 2 and 3 for clarity of interpretation of within-group changes in EDD in the presence of acute stressors.

In order to examine the effects of voluntary aerobic exercise on arterial resilience, we first confirmed that the voluntary wheel running intervention had similar effects on baseline endothelial function as have been reported previously [33]. We observed an age-related decline in carotid artery endothelial function, as peak endothelium-dependent dilation (EDD, Figure 1B) and EDD area under the curve (AUC, Figure 1C) were significantly lower in arteries of old control compared to young control mice. Consistent with our previous report [33], 10 weeks of voluntary wheel running late in life completely restored endothelial function in old animals to levels similar to those of young animals, whereas the exercise intervention had no further effect on endothelial function in arteries from young mice.

We also observed that the age-associated impairment in endothelial function was accompanied by a significant increase in basal arterial mitochondrial superoxide production, which was normalized by voluntary wheel running (Figure 1D). These results suggest that normalization of the age-related increase in arterial mitochondrial oxidative stress may contribute to improvements in arterial endothelial function induced by voluntary aerobic exercise.

### Voluntary aerobic exercise increases arterial resilience to acute mitochondria-specific stress

**Figure 2:**
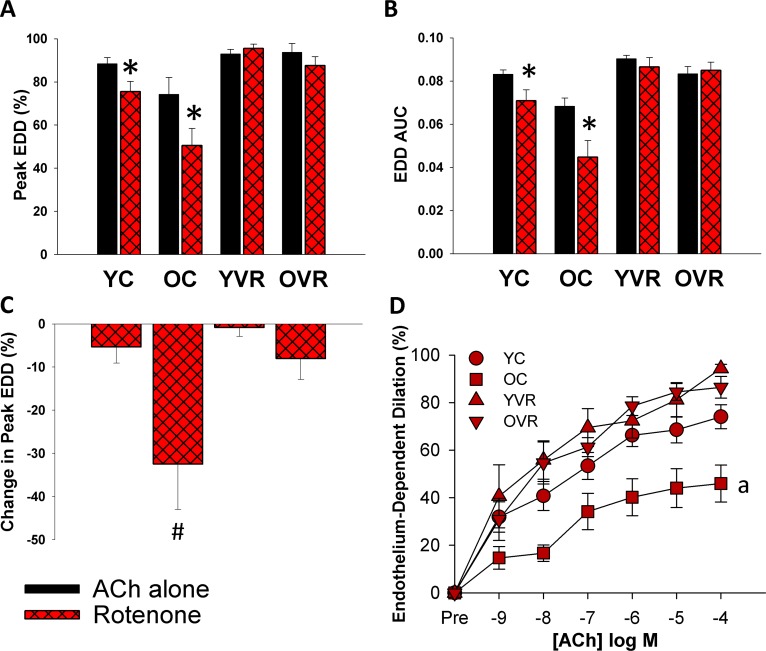
Voluntary aerobic exercise increases arterial resilience to acute mitochondria-specific stress (**A**) and (**B**) Peak endothelium-dependent dilation (EDD) and EDD AUC to acetylcholine (ACh) alone (black bars, n=10-12/group, shown again here for clarity) and in the presence of rotenone (red hashed bars, n=5-8/group) in carotid arteries from young control (YC), old control (OC), young voluntary wheel running (YVR) and old voluntary wheel running mice (OVR). (**C**) Relative reduction in peak EDD in the presence vs. absence of rotenone in arteries from YC, OC, YVR and OVR mice. (**D**) EDD dose-response curves to ACh in the acute presence of rotenone in carotid arteries from YC, OC, YVR and OVR mice. Data are presented as means with error bars representing SEM. * p<0.05 within-group versus ACh alone (repeated measures ANOVA), ^#^ p<0.05 vs. all other groups (one-way ANOVA), ^a^ p<0.05 relative change in EDD vs. all other groups.

Acute, *ex-vivo* treatment of arteries with a low concentration of mitochondrial Complex I inhibitor rotenone (0.5 μM for 40 minutes, [13]) slightly impaired peak EDD (Figure 2A) and EDD AUC (Figure 2B) in arteries of young control mice, whereas this acute mitochondrial stress caused substantial, further impairments in EDD in old control mice; importantly, the relative impairment in EDD in old control mice was significantly greater than in young control mice (Figure 2C). Voluntary wheel running improved arterial resilience to this stressor, such that rotenone had no significant effect in arteries of young or old voluntary wheel-running mice.

These results suggest that aging is accompanied by increased vulnerability of arteries to an acute mitochondria-specific stressor, and that voluntary aerobic exercise increases the ability of arteries to resist this stress.

### Voluntary aerobic exercise increases arterial resilience to simulated metabolic stress

**Figure 3:**
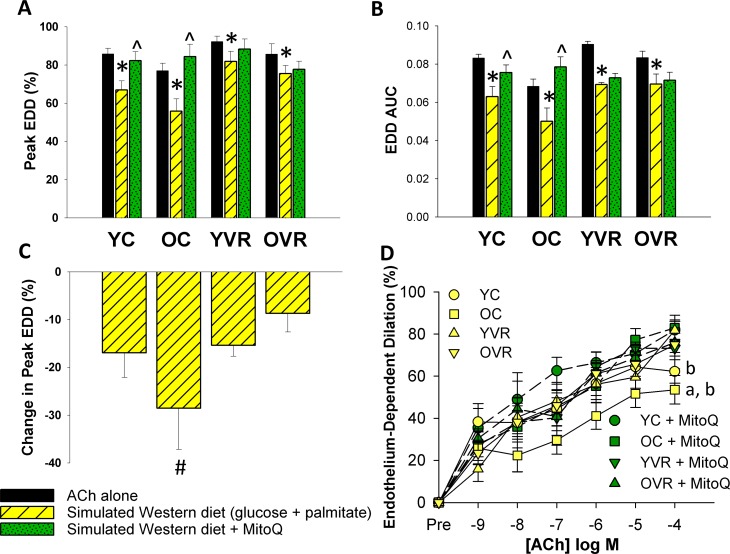
Voluntary aerobic exercise increases arterial resilience to acute simulated Western diet stress (**A**) and (**B**) Peak endothelium-dependent dilation (EDD) and EDD AUC to acetylcholine (ACh) alone (black bars, n=10-12/group, shown again here for clarity), in the presence of simulated Western diet (8 mM glucose + 160 μM palmitate; yellow hashed bars, n=4-8/group), and simulated Western diet + MitoQ (green hashed bars, n=4-8/group) in carotid arteries from young control (YC), old control (OC), young voluntary wheel running (YVR) and old voluntary wheel running mice (OVR). (**C**) Relative reduction in peak EDD in the presence vs. absence of simulated Western diet in arteries from YC, OC, YVR and OVR mice. (**D**) EDD dose-response curves to ACh in the acute presence of simulated Western diet (yellow symbols with solid lines) and simulated Western diet + MitoQ (green symbols with dashed lines) in carotid arteries from YC, OC, YVR and OVR mice. Data are presented as means with error bars representing SEM. * p<0.05 within-group versus ACh alone (repeated measures ANOVA), ^ p<0.05 within-group versus simulated Western diet (repeated measures ANOVA), ^#^ p<0.05 vs. all other groups (one-way ANOVA), ^a^ p<0.05 relative change in EDD vs. all other groups, ^b^ p<0.05 within-group vs. simulated Western diet alone.

Treating arteries acutely (40 minutes) *ex-vivo* with a simulated Western diet (8 mM glucose and 160 μM palmitate to simulate levels that might be present in the circulation following chronic consumption of a high-fat/high-sugar Western diet, [43]) reduced peak EDD (Figure 3A) and EDD AUC (Figure 3B) in arteries of both young and old control mice, but the degree of impairment was significantly greater in old control mice (Figure 3C). Simultaneous acute, *ex-vivo* treatment with the mitochondria-specific antioxidant MitoQ attenuated the simulated Western diet-induced reductions in EDD in young and old control mice, indicating that excessive mtROS contributed to the impairment induced by this stressor.

Voluntary wheel running improved arterial resilience to *ex-vivo* simulated Western diet (glucose and palmitate) in old mice, such that the reductions in peak EDD and EDD AUC induced by the simulated Western diet stressor in old exercising mice were less pronounced and not different in magnitude compared to those of young control and young exercising mice. In contrast to what we observed in arteries from young and old control mice, simultaneous *ex-vivo* MitoQ treatment had no effect compared to the simulated Western diet alone in either young or old exercising mice, suggesting an absence of excessive mtROS under conditions of simulated Western diet stress in these groups.

Together, these data suggest that, in sedentary mice, exposure of arteries to *ex-vivo* simulated Western diet (glucose and palmitate) induces or exacerbates endothelial dysfunction mediated by excess mtROS, and that voluntary aerobic exercise increases arterial resilience to the mtROS-associated component of this stressor.

### Acute ex-vivo stressors do not alter endothelium-independent dilation or vasoconstrictor tone

Following all assessments of EDD (response to ACh alone or in the presence of acute *ex-vivo* rotenone, simulated WD (glucose and palmitate) and/or MitoQ, as described above) endothelium-independent dilation (EID) was determined in response to exogenous application of sodium nitroprusside (SNP). Peak EID did not differ among groups (Table II), in line with previous studies [17, 33]. Additionally, the magnitude of vasoconstrictor tone elicited during pre-constriction with phenylephrine (PE) did not differ within individual arterial segments prior to versus following acute *ex-vivo* treatments (Table II). Together, these results indicate that the age-related differences in EDD and the transient stressor-related changes in EDD that we observed were not a result of differences in responsiveness of the vascular smooth muscle.

**Table II:** Endothelium-independent dilation and vasoconstrictor tone

		YC	OC	YVR	OVR
**Peak EID (%)**		90.1 (10.2)	88.1 (13.1)	92.7 (9.6)	95.3 (5.4)
**Preconstriction (%)**	Initial	.77 (.07)	.80 (.06)	.78 (.06)	.83 (.04)
	Before Rotenone	.78 (.07)	.84 (.03)	.77 (.04)	.85 (.02)
	After Rotenone	.79 (.05)	.83 (.07)	.77 (.06)	.84 (.03)
	Before P+G	.73 (.05)	.82 (.04)	.79 (.06)	.83 (.05)
	After P+G	.76 (.06)	.82 (.03)	.83 (.04)	.83 (.08)
	Before P+G+MitoQ	.76 (.13)	.83 (.04	.76 (.01)	.84 (.05)
	After P+G+MitoQ	.79 (.10)	.85 (.04)	.81 (.03)	.83 (.04)

Data are shown as mean (SD). Abbreviations: EID, endothelium-independent dilation assessed as magnitude of dilation in response to SNP in carotid arteries; YC, young control mice; OC, old control mice; YVR, young voluntary wheel running mice; OVR, old voluntary wheel running mice; P+G, simulated Western diet (*ex-vivo* glucose and palmitate).

Endothelium-independent dilation (EID) was assessed in carotid artery segments following all assessments of EDD to ACh in the absence and presence of acute *ex-vivo* stressors. Vasoconstrictor tone (pre-constriction) was quantified as the relative magnitude of constriction elicited by application of phenylephrine (2 μM, 5 min) immediately prior to each ACh dose-response. Pre-constriction was determined in all arterial segments prior to the first assessment of EDD (Initial, n=10-12/group) and then following acute *ex-vivo* application of rotenone (n=5-8/group), simulated Western diet (glucose + palmitate [P+G], n=4-8/group) and/or MitoQ (n=4-8/group). There were no significant within-group differences in EID nor in vasoconstrictor tone prior to versus following treatment with the acute *ex-vivo* modulators.

### Voluntary aerobic exercise normalizes age-related alterations in markers of arterial mitochondrial health but not respiratory protein content

**Figure 4:**
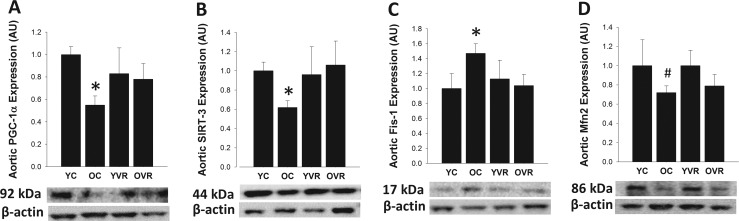
Voluntary aerobic exercise restores markers of arterial mitochondrial health in old mice Aortic protein expression of PGC-1α (**A**), SIRT-3 (**B**), Fis1 (**C**), and Mfn2 (**D**) in arteries from young control (YC), old control (OC), young voluntary wheel running (YVR) and old voluntary wheel running (OVR) mice. Representative images are presented below each panel with corresponding images of normalizer (beta actin) taken from the same region of the same blot. Data are presented normalized to beta actin and relative to the mean of the YC group as means with error bars representing SEM, n=6-8/group. * p<0.05 vs. YC, # p=0.1 vs. YC.

In order to gain insight into the potential effects of voluntary aerobic exercise on arterial mitochondrial health and antioxidant defenses, we assessed protein expression in homogenates of thoracic aorta, a large elastic artery (similar to the carotid) that also provides sufficient amount of tissue for analysis. [97] Aortic protein expression of PGC-1α and SIRT3, key markers of mitochondrial signaling and health, was significantly lower in old control compared to young control mice (Figure 4). Expression of Fis1, an important mediator of mitochondrial fission that is increased in settings of mitochondrial dysregulation [41, 44–46], was greater in arteries of old control versus young control mice, whereas expression of the key fusion mediator Mfn2 tended to be lower in arteries from old versus young control mice (p=0.1), indicating an age-associated shift in mitochondrial dynamics toward increased fission. Age-related differences in protein expression of a subset of these markers were comparable whether assessed in the carotid arteries or aorta (Table III), suggesting that arterial mitochondrial health changes similarly in these two large elastic arteries. Importantly, voluntary aerobic exercise in old mice normalized expression of PCG-1α, SIRT3 and Fis1 (with no effect on Mfn2), and had no further effect on expression of these proteins in young mice. These results suggest that voluntary aerobic exercise reverses age-related declines in markers of arterial mitochondrial health, perhaps contributing to some of its beneficial effects on endothelial function and stress resilience.

**Table III:** Protein expression of mitochondrial health markers in carotid arteries versus aorta of young and old mice

	YCar	OCar	YAor	OAor
PGC-1α (AU)	1.0 (0.14)	0.73 (0.15)	1.0 (0.12)	0.71 (0.15)
SIRT3 (AU)	1.0 (0.07)	0.70 (0.12)	1.00 (0.05)	0.80 (0.11)
Mfn2 (AU)	1.0 (0.33)	0.73 (0.35)	1.00 (0.36)	0.75 (0.18)

Data are presented normalized to beta actin and relative to the mean of the young group for each type of artery and are shown as mean (SEM), n=6/group. Abbreviations: YCar, carotid arteries from young control mice; OCar, carotid arteries from old control mice; YAor, aorta from young control mice, OAor, aorta from old control mice.

There were no statistically-significant differences in aortic expression of respiratory chain protein subunits from complexes I, II, III and V among groups (we were unable to resolve bands for the subunit of complex IV), indicating that arterial mitochondrial respiratory protein content was not altered with primary aging or voluntary aerobic exercise (Table IV).

**Table IV:** Aortic protein expression of mitochondrial respiratory complex proteins

	YC	OC	YVR	OVR
Complex I (AU)	1.0 (0.33)	1.04 (0.68)	0.81 (0.25)	1.08 (0.70)
Complex II (AU)	1.0 (0.43)	0.89 (0.54)	0.82 (0.31)	1.02 (0.69)
Complex III (AU)	1.0 (1.02)	1.02 (0.79)	0.80 (0.36)	0.99 (0.65)
Complex V (AU)	1.0 (0.49)	0.79 (0.34)	0.89 (.044)	0.93 (0.79)

Data are presented normalized to beta actin and relative to the mean of the young control group and are shown as means (SD), n=6-8/group. Abbreviations: YC, young control mice; OC, old control mice; YVR, young voluntary wheel running mice; OVR, old voluntary wheel running mice. No significant differences were detected among groups.

### Voluntary aerobic exercise augments markers of arterial antioxidant defense and stress response

**Figure 5:**
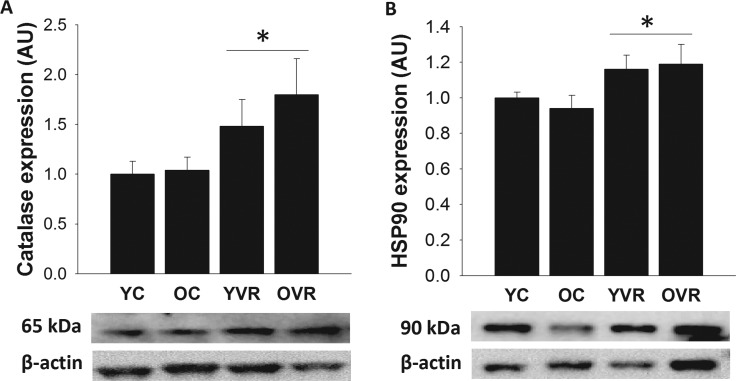
Voluntary aerobic exercise augments arterial markers of antioxidant defense and stress resistance Aortic protein expression of catalase (**A**) and Hsp90 (**B**) in arteries from young control (YC), old control (OC), young voluntary wheel running (YVR) and old voluntary wheel running (OVR) mice. Representative images are presented below each panel with corresponding images of normalizer (beta actin) taken from the same region of the same blot. Data are presented normalized to beta actin and relative to the mean of the YC group as means with error bars representing SEM, n=6-8/group. * p<0.05 vs. YC and OC (main effect of exercise).

Aortic protein expression values of the antioxidant protein catalase and stress-response protein Hsp90 were not different in old versus young control mice (Figure 5). However, voluntary aerobic exercise significantly increased expression of catalase and Hsp90 in arteries from both young and old mice. These results indicate that voluntary aerobic exercise augments these key mediators of cellular antioxidant defense and stress response [24, 47–48] in arteries, which may contribute to the improved resilience of arteries in exercising mice.

## DISCUSSION

The primary, novel findings of this study are that voluntary aerobic exercise improves resilience of aging arteries to acute, mitochondria-associated stress, and that this is accompanied by normalization of basal arterial mtROS production and improvements in arterial mitochondrial health and cellular antioxidant defense and stress response proteins.

Previous studies in our laboratory [17, 33] reported that late-life voluntary aerobic exercise restores baseline vascular endothelial function in old mice and we replicated this in the present study to confirm the effectiveness of the exercise intervention. These past studies identified normalization of arterial oxidative stress as a key mechanism underlying the beneficial effects of voluntary aerobic exercise. Here, we extend these findings by specifically examining the role of mitochondria-derived oxidative stress and its potential amelioration by voluntary aerobic exercise. In line with a recent study in our laboratory [13], we observed that age-related vascular endothelial dysfunction is accompanied by elevated arterial mitochondrial superoxide production. Importantly, we show here that voluntary aerobic exercise normalized mitochondrial superoxide production in arteries of old mice, suggesting that exercise-induced reductions in arterial mitochondrial oxidative stress may contribute to improvements in vascular endothelial function.

Our findings further extend previous work by demonstrating that, in addition to restoring baseline vascular endothelial function, voluntary aerobic exercise improves arterial resilience to acute stressors in old mice. Consistent with our previous report [13], we observed that acute treatment with rotenone, a mitochondrial Complex I inhibitor that can also induce mitochondrial superoxide production [49–50], impairs carotid artery endothelial function in old mice to a greater degree than arteries from young mice, indicating that aging arteries are more vulnerable to a mito-chondria-specific challenge. In the present study, we show that voluntary aerobic exercise completely restores the ability of aged arteries to withstand this acute mitochondrial stress.

Voluntary aerobic exercise also improved the resilience of arteries in response to a simulated Western diet stressor composed of *ex-vivo* glucose and palmitate, major nutrients present in the circulation with consumption of a high-fat, high-sugar Western diet. *Ex-vivo* simulated Western diet exposure caused a more substantial, further impairment in endothelial function in old sedentary mice versus young sedentary and young and old exercising mice. When considered from a translational perspective, although the *ex-vivo* simulated Western diet slightly impaired endothelial function in young sedentary and young and old exercising mice, the resulting level of function in these groups (peak EDD ~65-80%) was still substantially greater than that of old sedentary mice, in which the combined effects of aging and the *ex-vivo* simulated Western diet stressor resulted in dramatically reduced endothelial function (peak EDD ~45%).

The increase in arterial susceptibility to *ex-vivo* simulated Western diet with aging is similar to what is observed in a setting of chronic, *in vivo* Western diet consumption in mice. Old mice consuming Western diet for 10 weeks display significantly reduced endothelial function even compared to old mice consuming normal chow, and the magnitude of impairment induced by chronic Western diet consumption in old mice is much greater than the modest impairment observed in young mice consuming Western diet [17]. Simultaneous aerobic exercise, in the form of voluntary wheel running, completely preserves endothelial function in young and old mice chronically consuming Western diet [17]. In the present study, we observed that voluntary wheel running significantly improved arterial resilience to acute challenge with *ex-vivo* simulated Western diet, but did not confer complete protection against this stressor. This difference in exercise-induced arterial protection between studies may be due to the acute, *ex-vivo* nature of the simulated Western diet stress in the present study versus chronic Western diet consumption.

The observation that *ex-vivo* simulated Western diet exposure acutely impairs endothelial function is also consistent with clinical data that show reduced flow-mediated dilation following consumption of single high-fat meals [51–54] in heterogeneous populations that include older adults. Although data comparing arterial resilience in young versus older subjects is currently lacking, cross-sectional data support the premise that arterial resilience declines with age. Endothelial function is lower in older adults with moderately elevated fasting blood glucose or LDL cholesterol versus their age-matched sedentary peers with normal blood glucose or LDL levels [18–19], whereas—in support of our finding that exercise improves arterial resilience in mice—endothelial function is preserved at levels similar to young adults in older habitually exercising adults with both normal and elevated blood glucose or LDL cholesterol [18–19].

Simultaneously treating arteries from young and old sedentary mice with *ex-vivo* MitoQ abolished the impairment induced by simulated Western diet exposure, suggesting that the effects of this stressor were acutely mediated by excess mtROS. That the effects of this stressor may involve disruption of mitochondrial homeostasis is not unexpected, as glucose and palmitate independently increase mtROS production *in vitro* [55–62]. Additionally, arterial mitochondrial dysfunction is an important feature of metabolic diseases such as diabetes, in which arteries are chronically exposed to high circulating levels of glucose and lipids [44–45, 63].

Voluntary aerobic exercise conferred protection against *ex-vivo* simulated Western diet exposure in old mice, such that the impairment in endothelium-dependent dilation in arteries from old voluntary wheel running mice was much smaller in magnitude than that of old control mice, and not different from young control or young voluntary wheel running mice. This exercise-induced increase in arterial resilience appears to be primarily mediated by improved mitochondrial health, as simultaneous *ex-vivo* treatment with MitoQ had no effect on the response to simulated Western diet exposure in young or old exercising mice. However, the simulated Western diet did result in a slight reduction in EDD in both exercising groups that was unaffected by MitoQ, suggesting the involvement of additional mechanisms in the impairment induced by this stressor.

In line with normalization of basal mtROS production and increased arterial resilience to mtROS-mediated challenges, our results also demonstrate that voluntary aerobic exercise normalizes protein expression of key indicators of arterial mitochondrial health in old mice. Despite relatively low abundance [64] and minimal respiratory activity [10–12], arterial mitochondria play a vital role in maintaining arterial function, presumably via other roles involving intra- and extra-cellular signaling [10–12, 15]. However, arterial mitochondrial health and quality control decline with aging and in disease models of hypertension, NO deficiency, atherosclerosis, diabetes and metabolic syndrome [13–14, 27–29, 41, 44–45]. In the present study we observed age-related declines in PGC-1α and SIRT3, key regulators of mitochondrial biogenesis, health and antioxidant defenses [21–22, 39], as well as a shift in mitochondrial dynamics favoring increased mitochondrial fission (e.g., increased Fis1 and decreased Mfn2), a characteristic of mitochondrial dysfunction [41, 44–46]. Similar changes in these markers have been observed in models of disease and arterial dysfunction and with aging [28, 40–41]. It is well known that exercise improves mitochondrial health and signaling in tissues such as skeletal muscle, promoting increased mitochondrial quality [34–39]. Previous studies investigating the effects of exercise on arterial mitochondria indicate that although exercise induces favorable changes in arterial mitochondrial health in young, healthy animals, the arterial mitochondrial response to exercise is impaired with vascular and metabolic disease [27–29, 41]. One recent study reported increases in PGC-1α and mitochondrial respiratory protein content in aged rats following an exercise intervention [40]. Our observation that voluntary aerobic exercise normalized age-related changes in PGC-1α, SIRT-3 and Fis1 provide further evidence that arterial mitochondria of aged animals can adapt to exercise training and suggest that exercise-induced improvements in arterial mitochondrial health may be an important aspect of exercise-mediated vascular protection.

Finally, we also observed that voluntary aerobic exercise augments arterial expression of heat shock protein 90 and catalase in both young and old mice. Hsp are ubiquitously-expressed and highly-inducible chaperone proteins that are activated in response to a variety of cellular stressors [25, 47, 65] and interact with mitochondria to mediate cell survival pathways [24–25]. In the vasculature, Hsp90 interacts with endothelial nitric oxide synthase (eNOS) to promote NO production [66]. Exercise is a potent stimulus for induction of Hsp and increases localization of Hsp in the coronary and skeletal muscle vasculature [67–68], and this is thought to contribute to the protective effects of aerobic exercise in these tissues [67–69]. Although few studies have specifically examined the effects of aerobic exercise on Hsp induction in the systemic vasculature, resistance training-induced increases in femoral artery Hsp90 were associated with improve-ments in eNOS activity and arterial function in young and old rats [70]. In the present study, we observed that arterial expression of Hsp90 was significantly increased in both young and old mice following the voluntary aerobic exercise intervention.

Catalase is an antioxidant enzyme responsible for facilitating the decomposition of hydrogen peroxide, the dismutation product of superoxide, into water. Although superoxide is the primary species of ROS produced by mitochondria, the more stable hydrogen peroxide is thought to be the main transducer of many physiological and pathophysiological effects of mtROS [71–72]. Acute and chronic aerobic exercise training induce increases in catalase in the heart and vasculature and these adaptations are thought to contribute to reduced progression of vascular disease and increased cardiac resilience to acute ischemia/reperfusion injury [23, 73–75]. Additionally, previous studies from our laboratory and others have demonstrated increases in content and activity of other antioxidant enzymes, including the key mitochondrial antioxidant manganese superoxide dismutase, in arteries following aerobic exercise interventions [33, 76–77]. The exercise-induced increase in arterial catalase that we observed in this study is consistent both with previous reports and with our finding of increased resilience to mtROS-mediated acute stress. Our results suggest that aerobic exercise-induced augmentation of cellular stress response pathways and antioxidant defenses may contribute to vascular protective effects of voluntary aerobic exercise.

### Considerations and future directions

The primary aim of this study was to examine arterial resilience in response to acute stress and thus the stressors we employed in this study were applied to arteries *ex-vivo* for a short (~40 minutes) period of time. We designed our *ex-vivo* simulated Western diet to reflect two of the most prevalent nutrients (glucose and palmitate) that would be expected to be in contact with the vasculature in settings of chronic Western diet consumption and the concentrations used in the present study were selected to reflect levels commonly measured in the circulation of rodents ingesting Western-style diets [43, 78–80]. This stressor induced impairments in endothelial function, particularly in old sedentary mice, and we observed a similar age-related increase in susceptibility to simulated Western diet stress compared to chronic Western diet consumption [17]. However, we recognize that this simple model of *ex-vivo* challenge does not recapitulate all the elements of a Western diet and that acute stressors may not reflect all aspects of chronic challenges such as persistently elevated blood lipids or glucose or chronic consumption of a Western diet. For example, although excessive levels of mtROS contributed acutely to the stressor-induced dysfunction we observed in the arteries of sedentary mice, it is unlikely that the impairments were due to changes in mitochondrial DNA damage or gene expression, alterations that might be expected as a consequence of chronic mtROS elevations. Therefore, future studies are needed to examine arterial resilience to stress following *in vivo* acute and chronic Western diet consumption.

Although our results with acute MitoQ treatment indicate that suppression of endothelial function by the simulated WD stressor is primarily mediated by mtROS, there remained a small amount of impairment in arteries of the exercise-trained animals following acute MitoQ treatment, suggesting involvement of a non-mtROS mechanism. Previous work employing *ex-vivo* glucose and palmitate demonstrate that these compounds induce oxidative stress, a major source of which is increased production of mitochondria-derived superoxide [59–62, 81]. However, these compounds have also been reported to stimulate superoxide production via non-mitochondrial pathways, including activation of the pro-oxidant enzyme NADPH oxidase [82] or the renin-angiotensin system [83]. Experimental paradigms involving longer (e.g., 8-24 hour) exposures to hyperglycemia or palmitate suggest that these stressors can also induce changes in gene expression or post-transcriptional modifications, including suppression of AMPK signaling and induction of NFκ-B mediated pro-inflammatory signaling, that may act to suppress endothelial function [52, 84–86]. Importantly, our results indicate that voluntary aerobic exercise improves arterial resilience to the acute mitochondria-specific oxidative stress induced by simulated WD exposure.

In addition to the exercise-induced augmentation of antioxidant defenses we observed that may allow arteries to maintain vasodilatory function in the face of acute mtROS challenges, it is also important to consider the possibility that exercise may induce adaptations that enable arteries to dilate in response to mtROS, specifically hydrogen peroxide, which is emerging as a critical vasodilator in the coronary and resistance vasculature [87–91]. Age and exercise have been reported to influence the relative contribution of hydrogen peroxide to vasodilatory responses in resistance arterioles [75, 87, 91], and the exercise-induced enhancement of aortic catalase expression that we observed suggests an adaptation to elevated hydrogen peroxide levels. Thus, future studies are warranted to determine if hydrogen peroxide-mediated dilation may have contributed to the maintenance of vasodilation in the presence of acute mtROS stressors that we observed in the arteries from exercise-trained mice.

We observed improvements in protein markers of arterial mitochondrial health with voluntary aerobic exercise that are consistent with previous studies in young, healthy animals. However, in contrast to some previous reports investigating the effects of exercise on arterial mitochondria [27–28, 40–42] we did not observe an increase in arterial mitochondrial respiratory protein content with voluntary aerobic exercise. The lack of increase in mitochondrial protein content that we observed may be attributable to the nature of the exercise intervention, as voluntary wheel running—an intermittent stimulus—is distinctly different than the forced, continuous treadmill and swimming exercise paradigms employed in previous investigations. However, our results suggest that improvements in upstream regulators of arterial mitochondrial health induced by exercise can occur independently of changes in mitochondrial protein content and underscore the importance of mitochondrial quality control in arterial tissue. Indeed, the functional roles of vascular mitochondria may be more contingent on mitochondrial quality versus mitochondrial content. Mitochondria perform vital signaling functions in the vasculature [10–12] that depend on maintenance of an interconnected network of mitochondria via a balance of mitochondrial fission and fusion, as well as mitophagy [46, 92–93]. Thus, preservation of mitochondrial quality is critical in the vasculature, whereas an increase in mitochondrial respiratory protein content might be expected to confer little benefit to vascular cells that rely sparingly on aerobic metabolism. Although the exercise-induced improvements in markers of mitochondrial health we observed strongly suggest that voluntary aerobic exercise improves arterial mitochondrial function with aging, future work is needed to confirm whether the observed effects of aerobic exercise extend to more comprehensive indices of intact mitochondrial function in arteries.

We assessed markers of mitochondrial health in whole large elastic arteries and thus are not able to distinguish changes that may be specific to endothelial cells versus vascular smooth muscle cells (VSMCs). However, given that vascular mitochondria are particularly critical for intra- and inter- cellular signaling functions [10–12], mitochondria in both vascular cell types are likely important for regulation of arterial endothelial function. In addition to our finding that preserved arterial resilience with exercise is accompanied by improvements in whole artery mitochondrial health, previous work has also demonstrated that alterations in whole artery and VSMC mitochondrial health with aging, disease and exercise are accompanied by corresponding changes in endothelial function [13–14, 27–28, 40–42].

In conclusion, age-associated vascular endothelial dysfunction is accompanied by reductions in arterial resilience and mitochondrial health (elevated mtROS production, reduced markers of mitochondrial biogenesis/signaling, altered fission/fusion dynamics). Ten weeks of voluntary aerobic exercise improved arterial resilience to acute mitochondria-specific and *ex-vivo* simulated Western diet (glucose and palmitate) stressors, normalized age-related alterations in arterial mitochondrial health, and augmented arterial markers of antioxidant defense and cellular stress response. Overall, our results highlight the importance of healthy mitochondria for maintenance of arterial function and resilience with aging, and identify voluntary aerobic exercise as a later-life intervention that improves arterial resilience.

## METHODS

### Ethical approval

All experiments were approved by the Institutional Animal Care and Use Committee at the University of Colorado Boulder and conformed to the standards published in the Guide for Care and Use of Laboratory Animals (National Research Council, 2011).

### Animals and exercise intervention

Male c57Bl/6 mice were obtained from the colony at the National Institute on Aging (Bethesda, Maryland, U.S.) at ~5 or ~25 months of age. Animals were allowed to acclimate to our facility for 2 weeks, were kept on a 12-hour light:dark cycle and were provided normal rodent chow (Harlan 7019) and water ad libitum. Following the 2-week acclimation period, mice were randomly assigned to either a sedentary cage control group (young control [YC], n=11 and old control [OC], n=12) or to a voluntary aerobic exercise group (young voluntary wheel running [YVR], n=10 and old voluntary wheel running [OVR], n=11) for 10 weeks. The animals in the voluntary aerobic exercise groups were housed in cages with access to a running wheel (Lafayette Instruments, Lafayette, IN, USA) and were permitted to exercise ad libitum. Daily wheel running was monitored using Activity Wheel Monitor software (Lafayette, IN, USA) for 72 continuous hours once per week, and daily running activity was determined as the average distance run per 24 hour period. Following the 10-week intervention period, animals were euthanized and tissues and organs were harvested for assessment of arterial function and biochemical parameters as described below.

### Vascular endothelial function

Vascular endothelial function was assessed in isolated carotid arteries (2 per animal), as described previously [13, 94]. Measurement of carotid artery endothelial function in mice is an established model that reflects many features of age-associated arterial dysfunction in humans. [33, 97, 99] Briefly, arteries were dissected and cleared of surrounding tissue and cannulated onto glass micropipettes in a myograph chamber containing warmed (37 ºC) physiological saline solution. Arteries were pressurized to ~ 50mm Hg and allowed to equilibrate for 45 minutes. Following equilibration, the arteries were pre-constricted with 2 μM phenylephrine (Sigma Aldrich Corp., St. Louis, MO, USA) for 5 minutes, and endothelium-dependent dilation was assessed as the magnitude of dilation in response to increasing doses of acetylcholine (ACh, 1×10^−9^ – 1×10^−4^M, Sigma Aldrich Corp.). Following this assessment of baseline endothelial function, we next assessed endothelium-dependent dilation in the presence of acute *ex-vivo* stressors (as described below) in the same arterial segments. Following all measurements of endothelium-dependent dilation, endothelium-independent dilation in each segment was assessed as the magnitude of dilation in response to increasing doses of sodium nitroprusside (SNP, 1×10^−10^− 1×10^−4^ M, Sigma Aldrich Corp.), an exogenous NO donor. All vessel data are presented on a percentage basis to account for baseline differences in vessel diameter among animals. Peak EDD (greatest value of endothelium-dependent dilation) and the area under the dose-response curve (AUC, trapezoid method) were determined for each response.

#### Acute mitochondria-specific stress

To determine the effects of an acute mitochondria-specific stress on endothelial function, following assessment of dilation in response to ACh alone as described above, a sub-set of arteries (n = 7 (YC), 8 (OC), 5 (YVR), 6 (OVR)) was incubated with 0.5 μM rotenone (Sigma Aldrich Corp.), a mitochondrial respiratory Complex I inhibitor, for 40 minutes prior to pre-constriction followed by assessment of EDD to ACh, as previously described [13]. Low doses of rotenone have been shown to increase mitochondrial superoxide production from Complex I without completely inhibiting respiratory activity [49–50]. The rotenone-induced impairment in peak EDD was determined as the relative reduction in peak dilation in the presence versus absence of rotenone ([PeakEDD_ACh_-PeakEDD_Rotenone_/PeakEDD_ACh_]x100). Similarly, the rotenone-induced impairment in the EDD AUC was determined as the relative reduction in the AUC in the presence versus absence of rotenone ([AUC_ACh_-AUC_Rotenone_/AUC_ACh_]x100).

#### Acute ex-vivo simulated Western diet stress

To determine the effects of acute exposure to a more physiologically relevant stressor, we exposed a sub-set of arteries (n = 7 (YC), 8 (OC), 4 (YVR), 8 (OVR)) to an *ex-vivo*, simulated Western diet via intraluminal infusion for 40 minutes prior to pre-constriction followed by assessment of EDD to ACh. This *ex-vivo* challenge comprised warmed physiological saline containing 8mM glucose (in addition to 5mM glucose already present in physiological saline, Sigma Aldrich Corp.) and 300 μM palmitate (Sigma Aldrich Corp.), two of the major metabolites present upon consumption of a Western-style diet high in saturated fat and sugar. These concentrations were selected to simulate those reported in the circulation of rodents following chronic consumption of Western-style diets [43, 78–80]. The impairments in peak EDD and AUC induced by this *ex-vivo* simulated Western diet were determined as the relative reduction in peak EDD or AUC in the presence versus absence of *ex-vivo* simulated Western diet ([PeakEDD_ACh_-PeakEDD_WD_/PeakEDD_ACh_]x100; [AUC_ACh_-AUC_WD_/AUC_ACh_]x100).

#### MtROS mediation of ex-vivo simulated Western diet stress

To determine the role of mtROS in mediating the effects of the *ex-vivo* simulated Western diet, arteries were treated with the *ex-vivo* simulated Western diet (palmitate + glucose, as above) in the simultaneous acute, *ex-vivo* presence of the mitochondria-specific antioxidant MitoQ (1.0 μM, Antipodean Pharma-ceuticals, Inc., Menlo Park, CA, USA) to scavenge mtROS for 40 minutes prior to prec-constriction followed by assessment of EDD to ACh [13].

### Arterial mitochondrial superoxide production

Mitochondrial superoxide production was assessed using electron paramagnetic resonance spectroscopy, as previously described [13, 94–95]. Briefly, 2mm segments of thoracic aorta were dissected free of surrounding tissue and then incubated for one hour at 37º C in Krebs-HEPES buffer with the spin probe MitoTEMPO-H (0.5 mM, Enzo Life Sciences, Inc., Farmington, NY, USA), which specifically detects superoxide produced by mitochondria [96]. Following the incubation period, the amplitude of the signal was measured on an MS300 X-band EPR spectrometer (Magnettech GmbH, Berlin, Germany) with settings as follows: center field, 3350G; sweep, 80G; microwave modulation, 3000 mG; microwave attenuation, 7dB. Values are expressed relative to the mean of the young control group.

### Arterial protein expression

Because the carotid arteries, large elastic arteries, were used for *ex-vivo* measurement of endothelial function as described above, arterial protein expression was assessed using standard Western blotting procedures in homogenate from thoracic aorta, a representative large elastic artery, as performed previously [33, 97]. To confirm that aortic expression of our proteins of interest was similar to that of the carotid arteries, we assessed expression of a subset of proteins in both carotid arteries and aorta from a separate group of young and old animals (n=6/group). Following homogenization in RIPA buffer, 15 μg of protein were loaded into 4-12% polyacrylamide gels for separation by electrophoresis (Criterion System, Bio-Rad Laboratories, Inc., Hercules, CA, USA). Proteins were transferred onto nitrocellulose membranes (Trans-Blot Turbo System, Bio-Rad Laboratories, Inc., Hercules, CA, USA) and incubated overnight with the following primary antibodies: peroxisome proliferator-activated receptor gamma co-activator 1-alpha (PGC-1α, 1:1000, Novus Biologicals, USA), NAD-dependent deacetylase sirtuin-3 (SIRT-3, 1:500, AbCam, Inc.), catalase (1:1000, AbCam, Inc.) heat shock protein 90 (Hsp90, 1:1000, Enzo Life Sciences, Inc.), Total OXPHOS Rodent Antibody Cocktail (containing antibodies against Complex I subunit NDUFB8, Complex II –30k, Complex III Core protein 2, Complex IV subunit I, and Complex V alpha subunit; 1:250, Novus Biologicals, USA), Fis1 (TTC11; 1:500, Novus Biologicals, USA), Mitofusin 2 (Mfn2; 1:500, AbCam, Inc.), and beta actin (normalizer, 1:2000, Cell Signaling Technology, Inc.).

Proteins were visualized using horseradish peroxidase-conjugated secondary antibodies (Jackson ImmunoResearch Laboratories, Inc., Westgrove, PA, USA) and enhanced chemiluminescence (ECL) substrate (Pierce Biotechnology, Inc., Rockford, IL, USA) on a digital acquisition system (ChemiDoc-It; UVP, Inc., Upland, CA, USA). Individual protein expression values were quantified using Image J software (Bethesda, MD, USA) [98] and normalized to beta actin to control for differences in protein loading. Values for a single blot were expressed relative to the mean of the young control group. Western blots were run in duplicate and results for each animal were averaged. Representative images of individual proteins were obtained from the same blots using identical imaging conditions.

### Statistics

All statistical analyses were performed using IBM SPSS Statistics for Windows Version 22.0 (IBM Corp., Armonk, NY, USA). Data were assessed for the presence of outliers (Grubb's test), normality and homogeneity of variance prior to statistical analyses. Group differences were determined for the following variables using one-way ANOVA: Peak EDD and EDD AUC for each dose-response condition (ACh alone, rotenone, simulated WD, simulated WD+MitoQ); relative impairment induced by rotenone; relative impairment induced by simulated WD; arterial mitochondrial superoxide production; and arterial protein expression. Within-group differences in peak EDD, EDD AUC, EID and vasoconstrictor tone under different treatment conditions (e.g., in the presence vs. absence of MitoQ) were determined using a repeated-measures analysis of variance, with group as the between factor and treatment (simulated WD, simulated WD+MitoQ, etc.) as the repeated factor. When overall group or treatment differences were detected, specific pair-wise differences were identified with Fisher's least significant difference post-hoc tests (normally-distributed variables) or Games-Howell post-hoc tests (non-normally-distributed variables). P-values <0.05 were considered statistically significant. All data are presented as mean values (SD) in the text and mean values (SEM) in figures for clarity, unless otherwise indicated.

## Abbreviations

ACh: acetylcholine
CVD: cardiovascular diseases
EDD: endothelium-dependent dilation
eNOS: endothelial nitric oxide synthase
Hsp: heat shock protein
LDL: low-density lipoprotein
mtROS: mitochondria-derived reactive oxygen species
NO: nitric oxide
OC: old control mice
OVR: old voluntary wheel running mice
OXPHOS: oxidative phosphorylation
PGC-1α: peroxisome proliferator-activated receptor gamma co-activator 1-alpha
SIRT-3: NAD-dependent deacetylase sirtuin-3
SNP: sodium nitroprusside
YC: young control mice
YVR: young voluntary wheel running mice
